# Low LH level does not indicate poor IVF cycle outcomes with GnRh-a single trigger: a retrospective analysis

**DOI:** 10.1186/s12884-022-05251-4

**Published:** 2022-12-20

**Authors:** Xue-Fei Li, Qiao-Feng Wang, Qi-Qi He, Xue-Jiao Wang, Xing-Yu LV, Xiao-Jun Tang, Zhao-Hui Zhong, Yu-Bin Ding, Qi Wan

**Affiliations:** 1 The Reproductive Center, Chengdu Jinjiang Hospital for Women’s and Children’s Health, Sichuan 610041 Chengdu, China; 2 Joint International Research Laboratory of Reproduction and Development of the Ministry of Education of China, School of Public Health, Chongqing Medical University, 400016 Chongqing, China; 3 Department of Epidemiology, School of Public Health, Chongqing Medical University, Chongqing, China; 4 Department of Obstetrics and Gynecology, Women and Children’s Hospital of Chongqing Medical University, 401147 Chongqing, China

**Keywords:** IVF/ICSI, GnRH-a single trigger, LH levels, hCG retrigger

## Abstract

**Objective:**

To compare the in vitro fertilization/intracytoplasmic sperm injection (IVF/ICSI) cycle outcomes between patients with low and normal serum luteinizing hormone (LH) levels on the day after a gonadotropin-releasing hormone agonist (GnRH-a) single trigger. We further investigated the efficacy of human chorionic gonadotropin (hCG) retrigger on IVF cycle outcomes in patients with low LH levels after GnRH-a single trigger.

**Methods:**

We retrospectively analyzed 957 infertile patients (tubal factor, ovulation disorders, male sperm factor, or unexplained infertility) who were treated with IVF/ICSI at the Chengdu Xinan Gynecology Hospital from July 2017 to December 2020. Patients received sufficient GnRH-a single trigger were divided into two groups based on the serum LH levels on the next day of trigger: normal serum LH levels (≥ 10 mIU/mL) group (control group, n = 906) and low LH levels (< 10 mIU/mL) group (experimental group, n = 51). And the efficacy of hCG retrigger on IVF/ICSI cycle outcomes in 10 patients with low LH levels after GnRH-a single trigger.

**Results:**

There were no significant differences in IVF/ICSI cycle outcomes, including egg yield, two pronuclei fertilization rate, excellent embryo rate, or live birth rate of frozen-thawed embryos between patients with low and normal LH levels after GnRH-a trigger. It showed significantly higher risk of ovarian hyperstimulation syndrome in the group of low LH levels [ 0.7%(1/137) vs. 8.5%(4/47), *P* = 0.016] compared with the group of normal LH levels who received GnRH-a single trigger. The hCG retrigger had no obvious efficacy on cycle outcomes in patients with low LH levels, including oocytes retrieved, fertilization rate, embryo conditions, and live birth rate of frozen-thawed cycles.

**Conclusion:**

The IVF/ICSI cycle outcomes of patients with low LH levels on the day after GnRH-a administration were similar to those of patients with normal LH levels. Blood LH test might not be required on the day following the trigger. The hCG retrigger did not have any effect on the cycle outcomes, suggesting that immediate retriggering with hCG was unnecessary.

## Background

With the application of GnRH antagonist regimens for the prevention of a premature luteinizing hormone (LH) surge, the gonadotropin-releasing hormone agonist (GnRH-a) trigger was advocated as a valid alternative to human chorionic gonadotropin (hCG) trigger for controlled ovarian hyperstimulation in vitro fertilization/intracytoplasmic sperm injection (IVF/ICSI) cycle [1, 2]. Evidences have shown that oocyte maturation triggered by GnRH-a significantly reduce the risk of ovarian hyperstimulation syndrome (OHSS) compared with hCG trigger [3, 4]. However, the poor LH response to GnRH-a in some patients may cause adverse cycle outcomes in terms of the egg retrieval cycle and following embryo transfers cycle.

Serum LH < 15 mIU/mL on the morning after trigger has been defined as suboptimal response to GnRH-a trigger and reported to be significantly associated with an increased risk of empty follicle syndrome (EFS) and dramatically decreased oocyte recovery [5]. Theoretically, patients with poor post-trigger LH response, such as in the case of oocyte aspiration failure, hCG retrigger should be considered as an alternative regimen [5]. Another study suggested that LH levels measured at 12 h after trigger were not statistically significant with maturation rates and EFS was not reported [6]. However, there is still lack of more evidences to confirm the impacts of lower LH levels on IVF cycle outcome in patients who received GnRH trigger after GnRH antagonist-based stimulation protocols.

Therefore, it is crucial to further evaluate the relationship between serum LH level at 12h post-trigger and IVF cycle outcomes to find a safe and efficacious trigger medication for patients undergoing IVF therapy.

## Materials and methods

### Study design

Patients received IVF/ICSI at the Chengdu Xinan Gynecology Hospital for tubal factor, ovulation disorders, male sperm factor, or unexplained infertility from July 2017 to December 2020 were retrospectively analyzed. The inclusion criteria included age of 20–45 years, body mass index (BMI) of > 18 and < 30 kg/m^2^, the GnRH antagonist protocol was used for ovarian stimulation, and GnRH-a dabigatran (0.2 mg triptorelin acetate injection) was used alone as a trigger. The exclusion criteria were: cycles were triggered with hCG or dual trigger (GnRHa + hCG), patients diagnosed with hypothalamic amenorrhea or hypogonadotropic hypogonadism and uterine abnormality. This study was approved by the Ethics Committee of Chengdu Xinan Gynecology Hospital.

### Assisted reproductive methods

On the second or third day of menstruation, ovulation induction was initiated based on the patient’s number of antral follicles (AFC), follicle-stimulating hormone (FSH), LH, estrogen (E2), BMI and anti-Müllerian hormone (AMH) levels, with the appropriate dose of recombinant human follicle-stimulating hormone (r-FSH) or high-purity urinary follicle-stimulating hormone (HP-HMG). When most follicles were larger than 12 mm in diameter, or E2 was higher than 500 pg/ml, GnRH antagonist, Szczechoslovak (0.25 mg cetrorelic acetate for injection) or Olga (0.25ug Ganrik acetate injection) was administered. when the dominant follicles were larger than 18 mm in diameter, the GnRH-a Dabija (0.2 mg triptorelin acetate injection) was administered as the trigger. The trigger time was started at 9:00 p.m. on the hCG day, and egg collection was performed at 36–38 h after trigger. Blood was drawn at 13:00 on the next day of the trigger for testing serum LH levels. Serum LH < 10 mIU/ml at 16 h after a single trigger was defined as low levels. Subsequently, for patients with a poor response and low risk of OHSS, hCG (4000 IU or 10,000 IU) was administered immediately. Egg collection was arranged at around 12:00 of the next day. After egg collection, IVF or ICSI was performed according to the sperm quality. Owing to the high ovarian response in most patients, whole embryo freezing was performed to prevent the occurrence of late-onset OHSS, and only a small number of patients underwent fresh embryo transplantation. Therefore, the clinical outcomes of patients who underwent frozen-thawed embryo transplantation were analyzed.

### Statistical analysis

All statistical analyses were performed using SPSS 25.0 software. The Mann-Whitney U test was used for continuous data. Qualitative data were subjected to Fisher’s exact test or Chi-square test. Continuous variables were presented as median (interquartile range, IQR), and categorical variables were presented as cases (percentages) or percentages (cases).

Patients with an LH levels < 10 mIU/ml were taken in the experimental group, and patients with normal LH levels ≥ 10 mIU/mL were selected as the control group. The propensity score matching (PSM) analysis was used to reduce the potential confounding bias between the two groups. It makes the baseline characteristics of the two groups comparable. The matching variables included in the PSM were age, BMI, duration of infertility, infertility type, FSH, LH, E2, progesterone (P), AMH, AFC, gonadotropin (GN) dose, GN days, and fertilization method.

Propensity scores were calculated by logistic regression based on the above variables. Subsequently, patients with normal and low LH levels were matched in a 1:3 ratio by using the nearest neighbor matching method, and the caliper value was set to 0.2. Binary logistic regression and generalized linear models were used to analyze the impact of hCG supplementation on the embryo condition and pregnancy outcome of the patient. A *P* value < 0.05 indicated that the difference between the two groups was statistically significant.

## Results

### Basic characteristics of infertile women with different LH levels by using PSM

A total of 957 patients were included in the study, comprising 51 patients with LH level < 10 mIU/mL and 906 patients with LH level ≥ 10 mIU/mL after GnRH-a single trigger. The general comparison of patients with low LH levels (experimental group) and normal LH levels (control group) before and after PSM was listed in Table 1. No statistical difference was found in age, duration of infertility, or infertility type between the two groups before PSM. The BMI in the experimental group was significantly higher than that in the control group (*p* < 0.05), and basal LH was significantly lower than that in the control group (*p* < 0.05). After tendency scoring, no statistical difference was observed between the experimental and control groups in the basic characters.

**Table 1:** Comparison of the characteristics of infertile women received GnRH-a trigger after GnRH antagonist-based stimulation protocols via propensity score matching

Characteristics	Before propensity score matching		***P value***	After propensity score matching		***P value***
	**LH normal**	**LH low**		**LH normal**	**LH low**	
	***n = 906***	***n = 51***		***n = 137***	***n = 47***	
Woman’s age	29(27–32)	30(27–31)	0.555	29(27–31)	30(27–31)	0.843
BMI	21.48(19.53–23.84)	23.44(21.34–25.95)	**< 0.001**	22.96(20.945–25.91)	23.42(21.03–25.04)	0.935
Duration of infertility	3(2–5)	3(2–5)	0.612	3(2–5)	3(2–5)	0.841
Infertility type			0.452			0.594
Primary infertility	528(58.3)	27(52.9)		79(57.7)	25(53.2)	
Secondary infertility	378(41.7)	24(47.1)		58(42.3)	22(46.8)	
FSH	6.825(5.96–7.85)	5.62(5.06–6.91)	**< 0.001**	5.96(5.46–6.67)	5.98(5.13–6.96)	0.642
LH	5.46(3.92–7.93)	3.35(2.6–4.78)	**< 0.001**	4.16(2.99–5.49)	3.69(2.71–5.19)	0.215
E2	47(36–59)	44(32–54)	0.085	44(34–58)	42(32–53)	0.210
P	0.58(0.40–0.88)	0.50(0.27–0.76)	**0.042**	0.57(0.37–0.795)	0.50(0.29–0.81)	0.278
AMH	6.89(5.01–9.69)	6.35(4.64–9.82)	0.456	6.86(4.94–9.76)	6.47(4.66–10.21)	0.723
AFC	26(20–33)	23(17–30)	0.061	24(16.5–30)	24(18–30)	0.967
GN dose	1500(1350–1950)	2000(1575–2325)	**< 0.001**	1888(1500–2250)	1950(1538–2275)	0.460
GN days	10(9–10)	10(9–11)	**0.002**	10(9–11)	10(9–11)	0.808
Fertilization method			0.726			0.713
IVF	728(80.4)	42(82.4)		114(83.2)	38(80.9)	
ICSI	178(19.6)	9(17.6)		23(16.8)	9(19.1)	

Data are presented as median (IQR) or n (%)

*Abbreviations*: *BMI* body mass index, *FSH* follicular-stimulating hormone, *LH* luteinizing hormone, *E2* estradiol, *P* progesterone, *AMH* anti-Müllerian hormone, *AFC* antral follicle count, *GN* gonadotropin, *IVF* in vitro fertilization, *ICSI* intracytoplasmic sperm injection, *IQR* interquartile range

### Comparasion of clinical outcomes among infertile women before and after PSM

The comparison of ovulation induction and clinical outcomes of experimental group and control group before and after PSM was shown in Table 2. The P levels on the trigger day in the experimental group was higher than that in the control group (*P* = 0.015), while the E2 levels were lower in the experimental group than that in the control (*P* = 0.003). The incidence of OHSS was significantly higher in the experimental group compared with the control [ 0.7%(1/137) vs. 8.5%(4/47), *P* = 0.016]. However, there was no statistical difference between above two groups in terms of egg yield, two pronuclei (2PN) fertilization rate, excellent embryo rate, or live birth rate of frozen-thawed embryos.

**Table 2:** Comparison of baseline characteristics and IVF cycle outcomes between the two groups after the propensity score matching of patients received GnRH-a trigger after GnRH antagonist-based stimulation protocols

	LH normal (control group)	LH low (experimental group)	***P***-value
	***n = 137***	***n = 47***	
Trigger day’ P	1.56(1.22–2.25)	1.93(1.58–2.32)	**0.015**
Trigger day’ E2	7154(5691–8279)	5931(5310–6816)	**0.003**
Trigger day’ LH	1.79(1.00-2.95)	1.42(0.78–2.63)	0.124
Trigger day’ FSH	11.03(9.34–13.94)	11.15(9.04–13.91)	0.951
Number of follicles greater than 14 mm in diameter on Trigger day	19(16–23)	20(16–26)	0.259
Number of eggs obtained	18(15–25)	20(14–27)	0.364
Number of mature eggs	17(13–22)	18(12–25)	0.667
Number of 2PN fertilization	13(10–17)	13(8–19)	0.786
Cleavage embryo	5(2–7)	4(2–7)	0.828
Number of blastocysts formed	8(5–12)	7(4–13)	0.610
Incidence of OHSS	0.7(1/137)	8.5(4/47)	**0.016**
live birth rate of first FET	54.9(73/133)	55.6(25/45)	1.00
live birth rate of second FET	58.3(21/36)	45.5(5/11)	0.505
live birth rate of third FET	40.0(2/5)	60.0(3/5)	0.500

Data are presented as median (IQR) or % (n)

*Abbreviations*: *FSH* follicular-stimulating hormone, *LH* luteinizing hormone, *E2* estradiol, *P* progesterone, *2PN* two pronuclei, *OHSS* ovarian hyperstimulation syndrome, *FET* frozen thawed transplantation, *IVF* in vitro fertilization, *IQR* interquartile range

### Basic characteristics of low LH Levels in women with and without hCG supplementation

Patients with low LH levels after GnRH-a trigger were divided into two groups according to whether hCG retrigger was performed or not, and the baseline characteristics of the two groups were compared (Table 3). There were no significant differences in age, BMI, basal FSH, E2, P, GN dose, or GN days between two groups. However, the basal LH, AMH, and AFC of the GnRH-a single trigger group were significantly higher than those of the group with hCG retrigger .

**Table 3:** Comparison of the baseline characteristics of low LH level women with and without hCG retrigger

Characteristics	Retrigger	No retrigger	*P*-value
	***n = 10***	***n = 41***	
Woman's age	30(28-32)	30(27-31)	0.489
BMI	23.81(22.23-28.73)	23.42(20.98-25.49)	0.245
Duration of infertility	5(3-6)	3(2-5)	**0.048**
Infertility type			0.081
Primary infertility	8(80.0)	19(46.3)	
Secondary infertility	2(20.0)	22(53.7)	
FSH	5.29(4.48-6.19)	6.1(5.11-6.94)	0.226
LH	2.36(1.81-3.02)	3.93(2.76-5.77)	**0.003**
E2	47(42-62)	38(31-54)	0.221
P	0.57(0.26-0.85)	0.49(0.27-0.74)	0.561
AMH	5.30(2.93-6.20)	7.12(4.73-10.22)	**0.020**
AFC	14(11-19)	25(19-31)	**0.003**
GN dose	2025(1772-2700)	1950(1519-2325)	0.330
GN days	10(9-12)	10(9-11)	0.771
Fertilization method			0.061
IVF	6(60.0)	36(87.8)	
ICSI	4(40.0)	5(12.2)	

Data are presented as median (IQR) or n (%)

*Abbreviations*: *BMI* body mass index, *FSH* follicular-stimulating hormone, *LH* luteinizing hormone, *E2* estradiol, *P* progesterone, *AMH* anti-Müllerian hormone, *AFC* antral follicle count, *GN* gonadotropin, *IVF* in vitro fertilization, *ICSI* intracytoplasmic sperm injection; IQR, interquartile range

### IVF cycle outcomes of low LH levels women with and without hCG supplementation

The comparison of ovarian stimulation, embryo culture, and resuscitation transplant live yields in patients with low LH levels after GnRH-a single trigger with and without hCG rerigger was shown in Table 4. The number of follicles ≥ 14 mm on trigger day in the hCG retrigger group was significantly lower than GnRH-a single trigger group (*P* = 0.001), but there was no statistical difference in egg yield, 2PN fertilization rate, blastocyst rate, or live birth rate per resuscitation between the two groups. Moreover, regression analysis also confirmed that hCG retrigger had no significant effect on the egg retrieval rate, 2PN fertilization rate, excellent embryo rate, and live birth rate of frozen-thawed embryos of patients with LH levels below 10 mIU/mL after GnRH-a single trigger (Table 5).

**Table 4:** Comparison of ovarian stimulation, embryo culture, and resuscitation transplant live yields in women with low LH levels with and without hCG retriggering

	Retrigger	No retrigger	***P***-value
	*n*=10	*n*=41	
Trigger day’ P	1.55(0.93-2.07)	1.95(1.60-2.40)	0.490
Trigger day’ E2	5559(4862-6002)	5931(5588-7201)	0.144
Trigger day’ LH	1.78(0.95-4.29)	1.42(0.53-2.53)	0.196
Number of follicles greater than 14 mm in diameter on Trigger day	13(12-19)	21(18-27)	0.001
Number of eggs obtained	15(9-24)	20(14-28)	0.117
2PN fertilization number	9(6-15)	13(8-20)	0.120
High quality blastocyst	0(0-0)	0(0-1)	0.291
Incidence of OHSS	10.0(1/10)	9.8(4/41)	1.000
Live birth rate of first FET	60.0(24/40)	37.5(3/8)	0.272
Live birth rate of second FET	55.6(5/9)	0.0(0/3)	0.205
Live birth rate of third FET	75.0(3/4)	0.0(0/1)	0.400

Data are presented as median (IQR) or % (n)

*Abbreviations*: *FSH* follicular-stimulating hormone, *LH* luteinizing hormone, *E2* estradiol, *P* progesterone, *2PN* two pronuclei, *OHSS* ovarian hyperstimulation syndrome, *FET* frozen thawed transplantation, *IVF* in vitro fertilization, *IQR* interquartile range

**Table 5:** Effect of hCG retrigger on IVF cycle outcomes of patients with low LH level

**Outcome**	**Wald**	**OR(95%CI)/** **β** **(95%CI)**	***P*** **-value**
Live birth rate of first FET	0.581	0.482(0.074-3.15)	0.446
Live birth rate of second FET	0	/	/
Ovarian hyperstimulation syndrome (OHSS)	0	/	/
Number of eggs obtained	0.002	-0.130(-6.273-6.014)	0.967
Mature egg rate	1.064	-5.791(-16.793-5.21)	0.302
Fertilization rate	0.347	-4.243(-18.363-9.878)	0.556
2PN fertilization rate	0.001	0.182(-15.440-15.804)	0.982
Optimal embryo number at cleavage stage	0.363	-1.005(-4.275-2.265)	0.547
Excellent embryo number in blastocyst stage	0.007	-0.082(-2.021-1.856)	0.934
Number of blastocysts formed	1.265	-2.807(-7.698-2.085)	0.261
Number of blastocysts formed on D5	1.331	-2.232(-6.022-1.559)	0.249
Number of blastocysts formed on D6	0.173	-0.459(-2.619-1.701)	0.677
Number of blastocysts formed by high-quality embryo at cleavage stage	0.600	-1.240(-4.378-1.898)	0.439
The numb of frozen blastocysts from high-quality embryos at cleavage stage	0.738	-1.157(-3.798-1.483)	0.390

*Note*: Adjusted confounders included infertility duration, LH, AMH, and AFC. *OR* odds ratio, *CI* confidence interval

## Discussion

With the extensive application of the GnRH antagonist scheme, GnRH-a has been used as an alternative trigger drug, which can effectively promote the release of endogenous FSH and LH to induce follicle maturation and ovulation, as well as embryo development and pregnancy rates which are similar to those obtained using hCG trigger [7]. Compared with hCG trigger, the duration of the LH surge is shortened, thereby the risk of OHSS was reduced [8]. The incidence of EFS after a GnRH-a trigger has similar rate with hCG trigger [9–11]. Despite this, GnRH-a may still cause adverse reactions after the trigger, and relevant cases have been reported by many researchers [12–17].

The impacts of low serum LH levels (< 10 mIU/mL) of the day after GnRH-a trigger on IVF cycles outcome has not been clearly elucidated. Studies have reported decreased pregnancy rate after GnRH-a trigger, most likely related to low serum LH levels [6]. However, the present study showed that low serum LH levels did not exert adverse impacts on IVF cycle outcomes in terms of egg yield, 2PN fertilization rate, excellent embryo rate, and LBR (Table 2). We suggested that the primary reason for the differences among published studies might be due to the PSM used in our study which reduced the potential confounding bias between groups while other studies did not, and the second reason is the LH threshold used to characterize low LH level-patients was an arbitrary choice.

As mentioned, post-trigger suboptimal LH levels are correlated with an increased risk for EFS and a low oocyte retrieval rate. Various strategies supporting the luteal phase exogenously have been mplementated to achieve comparable pregnancy rates; however, some concerns for the effectiveness of GnRH-a to induce optimal response remain [5]. In this study, we tested whether hCG retrigger should be immediately used in when patients’ low LH detected after a GnRH-a single trigger. Unfortunately, our data did not show any efficacy in terms of egg yield, 2PN fertilization rate, or optimal embryo rate when hCG retrigger was applied to those patients with LH levels below 10 mIU/mL on the day after GnRH-a single trigger (Table 4). A similar data was also reported by Chang et al., no statistically significant difference in clinical outcomes between the cycles that were retriggered with hCG and successful GnRH-a triggers[18]. To summarize, hCG retrigger did not improve the clinical outcome regardless of whether LH reached the expected value. Hence, it is suggested that hCG retrigger is unnecessary under above circumstance.

Despite the fact that GnRH-a trigger prevents OHSS development, there are women diagnosed with OHSS who underwent ovarian stimulation for IVF using a long GnRH-a protocol [19]. In this study, our results showed that the incidence of OHSS in patients with low LH levels was significantly higher than that in patients with normal LH levels (Tables 2 and 4). Since previous studies have reported that the risk of OHSS was closely associated with more oocytes retrieved [20, 21], which may partly explain why the low LH group has a higher OHSS rate in our study. As shown in Table 2, patients with low LH levels seemed to obtain more oocytes than those patients with normal LH. More importantly, the higher incidence of OHSS in low LH group may result from the small sample size of these patients which increases the risk of type II error. This is one of the undeniable limitations of this study. Therefore, more well-designed randomized controlled trials are required to verify our results in future work.

In summary, this study did not show significant differences in IVF cycle outcomes, including egg yield, 2PN fertilization rate, excellent embryo rate, or live birth rate between patients with low and normal LH levels after GnRH-a trigger. The hCG retrigger on the next day had no obvious efficacy on IVF cycle outcomes in patients with low LH levels. However, patients with low LH response after GnRH-a trigger were a small probability event, with similar IVF cycle outcomes to those with normal levels of LH: among the more than 3,000 patients received GnRH trigger after GnRH antagonist-based stimulation protocols in our center over the past 4 years, only 51 cases had low LH response, and hence led to inadequate samples for analysis. What’s more, this study provides suggestions that in clinical practice, hCG trigger should be prioritized in reschedule egg retrieval for such patients.

## Data Availability

The datasets used and analysed during the current study are available from the corresponding author on reasonable request.
